# Dietary Supplementation of Brown Seaweed and/or Nucleotides Improved Shrimp Performance, Health Status and Cold-Tolerant Gene Expression of Juvenile Whiteleg Shrimp during the Winter Season

**DOI:** 10.3390/md19030175

**Published:** 2021-03-23

**Authors:** Mohamed Abdel-Rahim, Omar Bahattab, Fatma Nossir, Yahya Al-Awthan, Riad H. Khalil, Radi Mohamed

**Affiliations:** 1 Aquaculture Division, National Institute of Oceanography and Fisheries (NIOF), Cairo 21556, Egypt; mm.abdel-rahim@niof.sci.eg; 2 Biology Department, Faculty of Science, Tabuk University, P.O. Box 741, Tabuk 71491, Saudi Arabia; Obahattab@ut.edu.sa (O.B.); alawthan@ut.edu.sa (Y.A.-A.); 3 Department of Aquaculture, Faculty of Aquatic and Fisheries Sciences, Kafrelsheikh University, Kafrelsheikh 33516, Egypt; Fatma.fsh_0084@fsh.kfs.edu.eg; 4 Department of Biology, Faculty of Science, Ibb University, Ibb 70270, Yemen; 5 Department of Fish Diseases and Management, Faculty of Veterinary Medicine, Alexandria University, Alexandria 21544, Egypt; Riad.Khalil@alexu.edu.eg

**Keywords:** *Litopenaeus vannamei*, *Sargassum polycystum*, nucleotides, survival, growth, hemolymph, phagocytic activity, immune-related gene expression

## Abstract

This study was aimed to evaluate the efficiency of *Sargassum* *polycystum* and nucleotides- supplemented diets to improve immune response and cold-tolerance of juvenile *Litopenaeus vannamei*. Four treatments were evaluated: T1, the control, shrimp received only a basal diet; T2, a basal diet with 500 ppm nucleotides; T3, a basal diet with 500 ppm *S.* *polycystum* powdered; T4, a basal diet with 500 ppm nucleotides and 500 ppm *S.* *polycystum* powdered. Shrimp were fed experimental diets for 56 days. Results revealed shrimp fed T4 diet exhibited the best significant improvement in water quality, survival, growth, and feed utilization indices followed by T2, and T3, while T1 showed the worst values. Additionally, nonspecific immune responses (phagocytosis (%), lysozyme, phenoloxidase, super oxide dismutase (SOD) activity, total nitric oxide) were improved with 1.7–3.2-fold in T4 higher than T1. Histomorphology of hepatopancreas in T4 showed the most increased activation of the hepatic glandular duct system compared with the other treatments. Moreover, nucleotides/seaweed-supplemented diets upregulated relative expression of *cMnSOD*, *Penaeidin4*, and heat shock protein70 (*HSP70*) genes, while translationally controlled tumor protein *(TCTP)* was downregulated. In conclusion, the synergistic effects of both *S. polycystum* and nucleotides have many advantages as a growth promoter, immunostimulant, antimicrobial, and cold-tolerant stimulant to *L. vannamei*.

## 1. Introduction

Whiteleg shrimp, *Litopenaeus vannamei*, is one of the world’s largest farmed tropical shrimp species native to the Pacific Ocean [1]. The shrimp is characterized by rapid growth, high tolerance to a wide range of water salinity (5–40 ppt), high intensification, low protein requirements, and other features suitable for super intensive aquaculture [2]. Nevertheless, several environmental factors affect the shrimp growth and survival rates, such as water temperature [3], salinity fluctuation [4], sharp and rapid changes in pH [5], low dissolved oxygen [5], and toxins such as ammonia, nitrite, hydrogen sulfide, and heavy metals [6].

The worldwide progress of shrimp farming is facing rising environmental and pathological difficulties [7], besides low capability for cold water tolerance in most shrimp producing countries. Using natural immunostimulants as a healthier/safer therapy than administrating antibiotics and/or vaccines is recommended to control aquaculture pathogens [8]. Increasing shrimp immunity is an important direction in the current supporting policies in order to prevent shrimp diseases [7].

Nucleotides are chemical compounds that played chief roles in nearly all biological cellular metabolic processes like energy production (e.g., ATP), metabolism, cell signaling (e.g., cAMP). They provided the natural building block of DNA, RNA, and essential cofactors in the biosynthesis of proteins and enzymes (e.g., NAD+) [9,10]. It is supposed that crustaceans have inadequate ability to synthesize nucleotides de novo. Therefore, it would be highly beneficial to support shrimp feeds with dietary supplementation of nucleotides [11], especially during the stressful periods. Though nucleotides’ usage is relatively novel in shrimp aquaculture, there is growing evidence exhibiting their benefits [12].

Marine plants (seaweeds or macroalgae) are considered a rich source of bioactive compounds [13,14]. There is a rising awareness of the exploitation of seaweeds (dried, isolated compounds and/or extracts) as an aquafeed additive in shrimp farming. Seaweeds (red, brown, and green) are characterized by many properties such as nutritional, growth-promoting, cytostatic, antioxidant, immune-modulatory, neuroprotective, hepatoprotective, antibacterial, antifungal, and antiviral activities [15,16,17,18,19].

The seaweed genus, *Sargassum* sp. is the most important, common, and widespread brown seaweed that is found in tropical and subtropical regions. They have been used in agriculture, the food/feed industry, folk remedy, and cosmetology [17,20]. Various Sargassum species have folk applications in human food as a rich source of proteins, carotenoids, vitamins, and minerals [17]. *Sargassum* sp. has ecological and economic importance in producing nutritional feed additives and different medicinal products. Many species are found in Egypt; some were identified, and many more need to be identified [21]. *Sargassum polycystum* C. Agardh is brown seaweed abundant in the Red Sea, Hurghada, Egypt. *S. polycystum* (sp.) is traditionally used against several human diseases [22].

The biochemical composition of *S. polycystum* revealed the nutritive value of crude protein (14.2%), crude lipid (7.6%), fiber (21.3%), carbohydrate (25.0%), polyunsaturated fatty acids, soluble nutritional fibers, and ash (29.0%) higher than that documented in terrestrial plants and animal products [23]. Sargassum is the richest source of fucoidans and alginic acids [24]. Several bioactive compounds were extracted from different Sargassum species as polysaccharides, alkaloids, tannins, saponins, flavonoids, glycose, sulfated polyphenols, phenolic, sterols, B-carotene, terpenoids, sargaquinoic acids, sargachromenol, and pheophytin [17,25,26]. Chotigeat et al. [27] stated that the extracts from several brown algae species containing a polysaccharide element had an effective capability to improve the disease resistance and immune responses of different aquatic species. In addition, alginic acid [28] and fucoidan [29] extracted from brown algae showed a useful role as an immune induction in preventing various aquatic diseases [30]. The immunity parameters, such as total hemocyte count and phagocytic activity, are well-documented [31,32].

The winter months in Egypt and many other Mediterranean countries (December, January, and February), with an average day/night difference of 10–16 °C, are the main drawback for continuous aquaculture production of shrimps. The cold winter caused massive economic death to the *L. vannamei* aquaculture sector. However, insignificant data is available about shrimp responses during cold temperature stress. In the present study, the effects of nutritional additives on the cold tolerance of whiteleg shrimp were studied. Accordingly, this study investigated the single and synergistic effects of dietary supplementation of brown seaweed, *S. polycystum,* and nucleotides (NucleoforceFish™) on water quality, growth, feed utilization, proximate composition, immune responses, phagocytic activity, histology of hepatopancreas, and intestinal tract, besides antioxidant, antimicrobial, and cold-tolerant gene expression in juvenile whiteleg shrimp, during the winter season. The results could deliver valuable orientation towards the importance of dietary management through the use of feed additives as a potential method to adapt to or face the winter season.

## 2. Results

### 2.1. Water Quality Parameters

The water quality parameters in experimental tanks, including salinity, water temperature, pH, dissolved oxygen, total ammonia nitrogen, and unionized ammonia, are illustrated in Table 1. There were no significant (*p* > 0.05) differences in the findings of salinity and temperature. The nutritional additives improved significantly (*p* ≤ 0.05) the dissolved oxygen level in the experimental tanks with the highest elevation being recorded in T4. Both TAN and NH3 were decreased significantly with the nutritional additives, with the lowest significant values being recorded in T4 (*p* ≤ 0.05).

### 2.2. Survival, Growth, and Condition Factor

Growth performance indices (final weight, weight gain “WG”, average daily gain “ADG”, and specific growth rate “SGR”), condition factor, and survival of whiteleg shrimp fed nucleotides- and *S. polycystum*-supplemented diets are presented in Table 2. The supplemented diets had significant (*p* ≤ 0.05) effects on the previously tested parameters with the best results being observed in shrimp fed T4. The ADG improved by 10.3%, 5.2%, and 17.2% in the T2, T3, and T4 treatments, respectively, compared with the T1 treatment. In the same direction, the survival rate improved by 10.0%, 6.7%, and 12.2% in the T2, T3, and T4 treatments in respect to the T1 treatment, respectively. The data for condition factors were consistent with the growth and survival findings with the highest value being observed in T4.

### 2.3. Feed Utilization Indices and Whole-Body Proximate Composition

The feed conversion ratio (FCR), protein efficiency ratio (PER), protein productive value (PPV), and energy utilization (EU) data are illustrated in Table 2. The feed utilization indices improved significantly (*p* ≤ 0.05) with *S. polycystum* and nucleotides’ dietary supplementation. Shrimp that received T4 presented significantly (*p* ≤ 0.05) better FCR, PER, PPV, and EU values than those raised at T1, T2, and T3. The best value of FCR was achieved for the T4 treatment (2.78), while the worst one was for T1 (3.91) treatment. The FCR, PER, PPV, and EU were reduced by 28.9% and improved by 40.8%, 40.0%, and 31.3% at T4 treatment than those recorded at T1, respectively. The effects of *S. polycystum*- and nucleotides-supplemented diets on the whole-body proximate composition are illustrated in Table 2. Dry matter and fiber content increased significantly (*p* ≤ 0.05) in diets T2, T3, and T4 compared with T1. The lowest retention of protein, ether extract, ash, and carcass energy were observed at the diet supplemented with *S. polycystum* (T3). However, the higher content of both protein and carcass energy were recorded in T1, and T4, with no significant (*p* > 0.05) differences between both.

### 2.4. Nonspecific Immune Responses

The nonspecific immune parameters, including total hematocytes count, phagocytic index, total protein, lysozyme activity, phenoloxidase activity, superoxide dismutase activity, and total nitric oxide of T4 shrimp, were significantly (*p* ≤ 0.05) higher than the other treatments (Table 3). Nucleotides- and *Sargassum*-supplemented diets (T2, T3, and T4) significantly (*p* ≤ 0.05) increased immune parameters in respect to the control (T1). Nucleotides-supplemented diet showed better significant (*p* ≤ 0.05) results compared to *Sargassum*-supplemented diet. Diet 4 with a mixture of 500 mg/kg nucleotides and 500 mg/kg *S. polycystum* powdered showed 1.5–3.2-fold higher immune parameters when compared with the control one.

### 2.5. Phagocytic Activity and Phagocytic Index

Phagocytic activity and phagocytic index were significantly (*p* ≤ 0.05) raised in T2, T3, and T4 compared to the control treatment (Table 3). Treatments T3 and T4 exhibited a developed phagocytic activity when compared with the control one (T1). However, T4, T2 and T3 showed higher phagocytic index with respect to T1.

### 2.6. Histological Results

**Figure 1:**
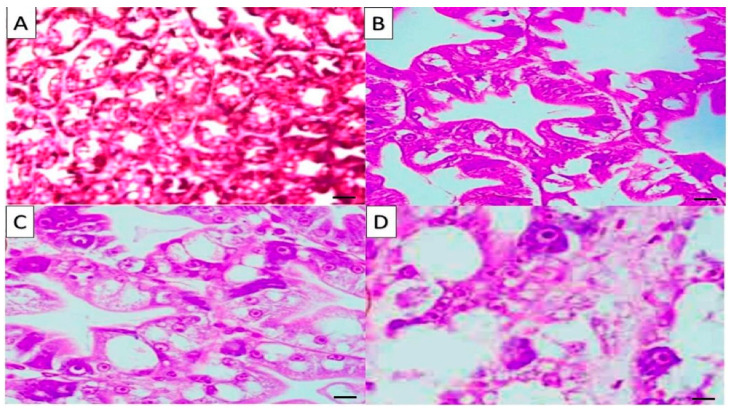
Hepatopancreas (HP) of *Penaeus vannamei*: (**A**) T1 showed normal HP structure and tubule epithelial cells surrounded by hemolytic infiltration; (**B**) T2 showed slight hemocyte infiltration, and normal hepatopancreas lumen and tubule, (**C**) T3 showed mild hemocyte infiltration and normal hepatopancreas lumen and tubule; (**D**) T4 showed highly activation of the hepatic glandular duct system. H&E stain magnification (×200), bar = 50 µm.

Figure 1 shows the histology of the hepatopancreas (HP) of *L. vannamei*. In T1, a normal HP structure and tubule epithelial cells surrounded by hemolytic infiltration were observed, while in the T2, the hepatopancreas showed slight haemocyte infiltration and normal hepatopancreas lumen and tubule. In T3, the hepatopancreas of *Penaeus vannamei* displayed mild haemocyte infiltration and normal hepatopancreas lumen and tubule, whereas T4 showed high activation of the hepatic glandular duct system.

**Figure 2:**
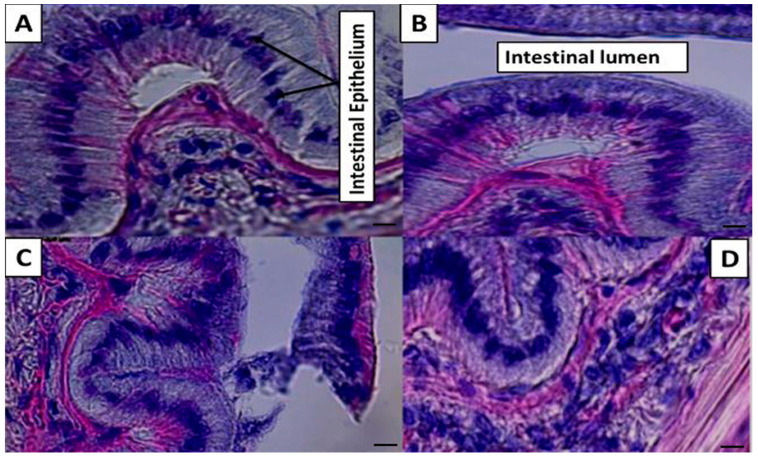
Histology of the intestinal tract of *P. vannamei:* (**A**) T1 showed the intestinal epithelium presented intact, developed, organized, and well-defined cells, as well as the absence of vacuoles and intercellular spaces; (**B**) T2 showed the intestinal epithelium and intestinal lumen increase in cells and width compared to T1 and intestinal lumen; (**C**) T3 showed the highest record of the intestinal epithelium and intestinal lumen compared to T1 and T2; (**D**) T4 showed the intestinal epithelium and intestinal lumen increase in cells and width compared to T1 and T2. H&E stain magnification (×200), bar = 50 µm.

The histology of the intestinal tract of *P. vannamei* is illustrated in Figure 2. In the T1, the intestinal epithelium showed intact, developed, organized, and well-defined cells, in addition to the absence of vacuoles and intercellular spaces, while in the T2, the intestinal epithelium and intestinal lumen showed an increase in cells and width compared to T1. The intestinal tract of *P. vannamei* in T3 showed the highest record of the intestinal epithelium and intestinal lumen compared to T1 and T2, whereas T4 showed an increase in cells and width of the intestinal epithelium and intestinal lumen compared to T1 and T2 positively.

### 2.7. Bioassay of Four Immune-Related Genes in Shrimp P. vannamei

**Figure 3:**
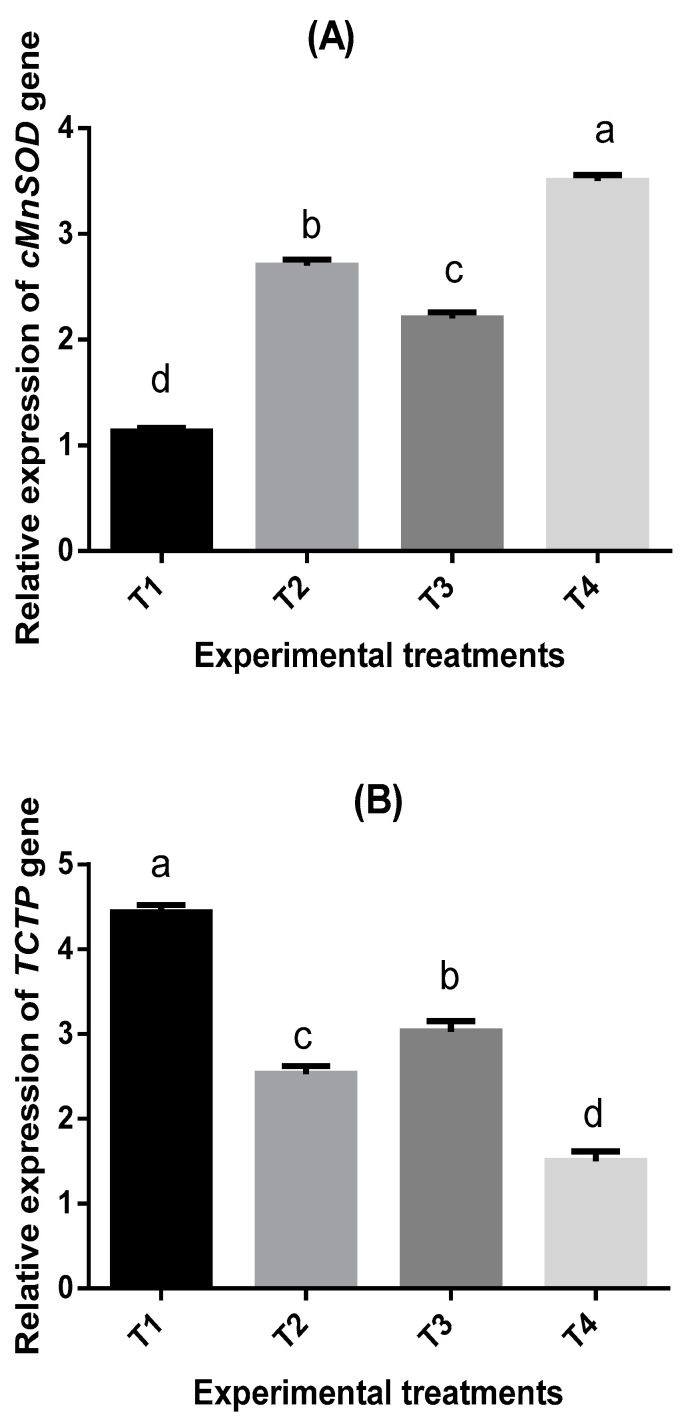

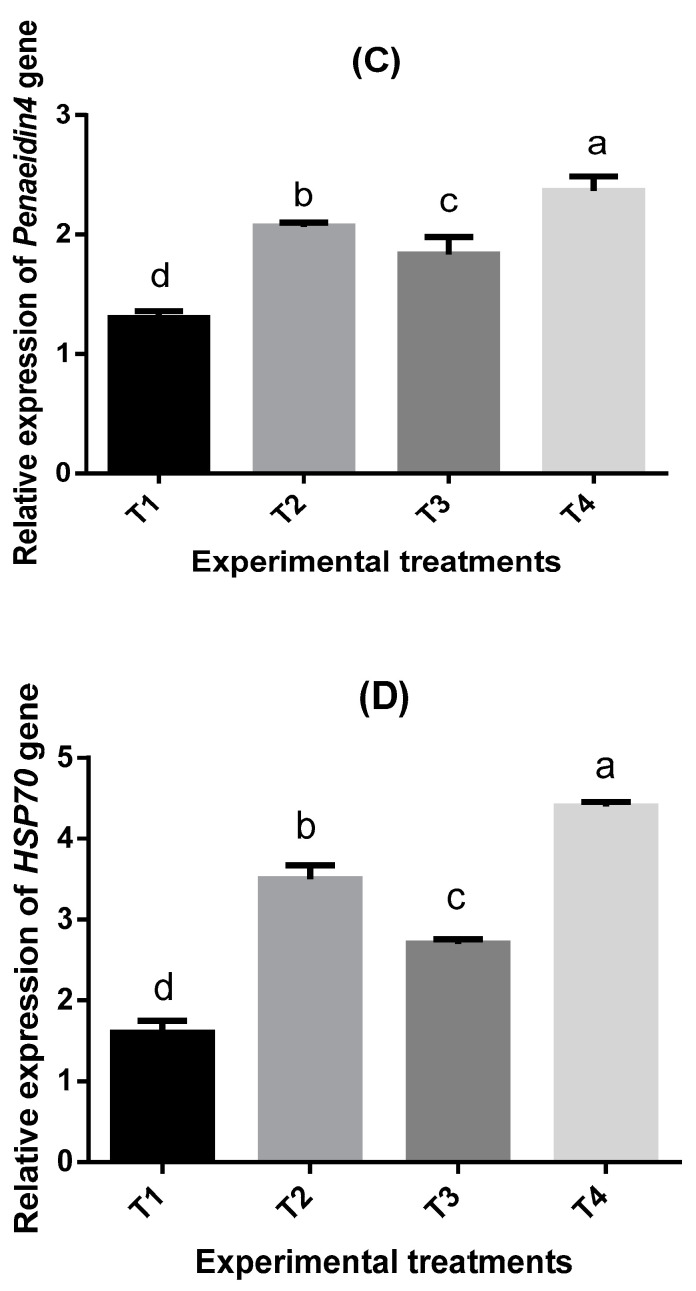
Relative expression of the cytosolic manganese superoxide dismutase (cMnSOD) gene (**A**), translationally controlled tumor protein (TCTP) gene (**B**), a novel antimicrobial peptide Penaeidin4 gene (**C**), and heat shock protein70 (HSP70) gene (**D**) in hemocytes of *P. vannamei* fed with nucleotides- and *Sargassum polycystum*-supplemented diets (T1, T2, T3, and T4). Sampling (*n* = 3) relative expression was calculated with the equation RQ target/geometric mean of RQ reference genes. Reference genes: β-actin. Results are presented as mean ± SE. Different letters (a–d) indicate significant differences (*p* < 0.05).

To determine the transcriptional responses of *P. vannamei* to *S. polycystum-* and nucleotides-supplemented diets, the authors assessed mRNA expression of four immune-related genes. The *cMnSOD*, translationally controlled tumor protein (*TCTP*), *penaeidin4*, and heat shock protein70 (*HSP70*) gene expressions were modulated, revealing different treatment influences, as shown in Figure 3A–D. The mRNA expression of *cMnSOD* and *penaeidin4* genes was significantly upregulated. The mRNA expression of the *cMnSOD* gene was upregulated significantly (*p* ≤ 0.05) after feeding shrimp with T2, T3, and T4 (Figure 3A), but the reduction of its expression to the control level (T1) was observed. The relative expression of *cMnSOD* was 3.1-fold higher in T4 than T1. Conversely, the mRNA expression of the *TCTP* gene was significantly downregulated (*p* ≤ 0.05) after feeding shrimp with T2, T3, and T4 (Figure 3B). The mRNA expression of *penaeidin4* was upregulated significantly (*p* ≤ 0.05) after feeding shrimp with T2, T3, and T4, and the reduction of its expression to the control level was observed in T1 (Figure 3C). The dietary treatments significantly (*p* ≤ 0.05) upregulated the expression of *HSP70* after feeding shrimp with T2, T3, and T4, but the reduction of its expression to the control level was observed inT1 (Figure 3D). The relative expression of *HSP70* gene was 2.75-fold higher in T4 than T1.

## 3. Discussion

Modern feed additives and their impact on improving water quality, survival rates, feed utilization, health status, and disease resistance of cultured aquatic organisms are critical issues that scientific research focused on during recent decades. The direct/indirect effects of dietary nucleotides and seaweed on water quality have not been focused on much before. The present study revealed a significant decrease in both TAN and NH3, parallel with the increase in dissolved oxygen concentration. This might be attributed to the better utilization of offered feeds in the diets T2, T3, and T4, compared with T1. The minimum averages of water temperature recorded in this study were higher than the lethal limits stated by [33]. They found that the cold tolerance level of *L. vannamei* varied between 7.5 and 11 °C and it seems to be more sensitive to low water temperatures than other penaeid species. Additionally, the previous authors stated that for successful commercial aquaculture, water temperature must never fall below12 °C during the overwintering season to avoid mortalities.

The indirect effects of marine algae on water quality in shrimp ponds are illustrated by [34], who tested the effects of coculture of two species of macroalgae separately (*Gracilaria vermiculophylla* and *Dictyota dichotoma*) in *L. vannamei* ponds, and recorded a significant decrease in the concentration of both ammonia and nitrites.

The growth rate of a living organism is a measure of the extent of utilization of the feed provided. In the present study, it was found that the experimental diets containing *S. polycystum* and nucleotides induced a clear improvement in growth performance, especially the T4 treatment receiving both additives. This result is in agreement with [35] concerning *Sargassum* addition in *M. rosenbergii*, and [9,12,36,37], regarding the addition of the nucleotide. Arizo et al. [35] concluded that dietary supplementation of fucoidan’s extracted from *S. polycystum* at 500 mg kg^−1^ exhibited the highest increment on growth performance parameters. The *P. monodon*, PL15–35 fed *Artemia instar* II enriched with various concentrations of *Sargassum sp.* extracts at 250, 500, and 750 mg/L showed significantly higher growth performance indices than the control group; the best concentration was 750 mg/L [38]. Moreover, *Penaeus indicus* juveniles fed with *Sargassum wightii*-enriched *Artemia nauplii* exhibited higher weight gains and SGR than the control after 30 days of feeding trial [39].

For nucleotides, Xiong et al. [40] found that shrimp, *L. vannamei* fed 50 g/kg nucleotides-enriched diet displayed significant higher growth rates compared to nonsupplemental diet. Similarly, Lucien-Brun and Vidal [10] stated that shrimp fed the nucleotides-supplemented feed had 23.2–25.0% higher final weight than the control group in normal condition and 57.7% after infection with white spot syndrome virus. Andrino et al. [37] recorded higher values in shrimp gain and growth indices of *L. vanemmei* fed with a nucleotide-supplemented diet than those fed the control diet. However, Schleder et al. [18] did not find a significant effect of *S. filipendula* dry biomass’ dietary addition on the growth performance of Pacific white shrimp at concentrations 0.5–4%. The effect of seaweed on the performance of shrimp was significantly different. It depended on the species used, the processing technique of the extract, the quality of feed, the content of fishmeal in the diet, and the inclusion level [18,41,42].

The lower FCR, the more effectively fish utilizes the aquafeed nutrients [43]. In the present study, the decrease in the FCR values obtained in the *Sargassum*-enriched diet (T3) and nucleotides-enriched diet (T2) is consistent with the obtained results by [35,37], respectively. Arizo et al. [35] stated that fucoidan’s dietary addition extracted from *S. polycystum* at 500 mg kg^−1^ recorded the lowest values of FCR. Schleder et al. [18] noted that seaweed’s dietary supplementation in the shrimp diet improved nutrient absorption, feed utilization, and digestive tract development. Additionally, Chithambaran and David [44] found that plant extracts stimulated shrimp digestive enzymes. Furthermore, nucleotide-fed crustaceans exhibited better FCRs and more efficient utilization of proteins and lipids [37,40]. Xiong et al. [40] indicated that shrimp fed 50 g/kg nucleotides-enriched diet showed significantly higher PER, PPV, and lower FCR than a nonsupplemental diet. Similarly, Lucien-Brun and Vidal [10] recorded that shrimp fed the nucleotides-supplemented feed had 27.3% better FCR than the control one.

For the present study’s survival rate, nutritionally enriched groups increased survival rate compared to the control one. This is consistent with the other researchers regarding *Sargassum* additives [35] and nucleotides [9]. This might be attributed to the fact that nucleotides/*Sargassum* supplements improved innate and adaptive immune systems, reducing pathogenic infections [9], and tolerated more efficiently cold-water temperatures as recorded in the present study. Shrimp-fed nucleotides-enriched diet exhibited higher survival rates during different normal growing stages [45,46] and violent environmental changes conditions like changes in salinities [47]. Similarly, Lucien-Brun and Vidal [10] recorded that shrimp fed the nucleotides-supplemented feed had a 14.3–38.5% higher survival than the control and 63% higher after infection with white spot syndrome virus.

In the present study, whole-body proximate composition indicated a significant decrease in lipid and protein content in shrimp fed *Sargassum* and nucleotides diets. In *Sargassum* fed group, this might be attributed to the high level of amylase activity and the lower activity of the proteolytic enzymes, as stated by [18] who found that the dietary addition of seaweeds, *S. filipendula* did not increase proteolytic enzymes but increased amylase activity significantly. The *S. polycystum* extracts have hepatoprotective and antioxidant properties, enhancing insulin sensitivity in diabetic rats [48]. The low doses of *S. polycystum* extract at 150 mg/kg body weight were useful in improving histological damages in diabetic tissues and organs. In comparison, the concentration of 300 mg/kg body weight was helpful to the pancreas but may be poisonous to the kidney and liver of diabetic rats [48]. Therefore, with higher insulin content, a direct relation between the lower content of lipids and the dietary inclusion of *Sargassum* might be explained. For nucleotides fed diet, the authors of [40] found no significant differences in the whole body and proximate muscle composition of *L. vannamei* fed nucleotides-rich yeast diets at doses of 10 and 30 g/kg. However, the higher dose of 50 g/kg increased the content of protein significantly. Abedian-Kenari and Oujifard [49] found that dietary nucleotides did not significantly change the chemical composition (moisture, protein, SFA, MUFA, PUFA, and ash) in *L. vannamei*. Similar results were observed in white shrimp [50].

The nutritional quality of shrimp diets positively or negatively affects the morphology, amelioration, and functional health status of the gastrointestinal (GI) tract [40,50]. In the present study, the dietary inclusion of both *Sargassum* and nucleotides improved the intestinal epithelium histology and lumen with more cells and width. The current study results are alike with earlier studies for shrimp [12,40,50] regarding the positive effects of nucleotides. Dietary nucleotides have a constructive impact on intestinal growth [12], intestinal villi height, and jejunum wall thickness [50]. This development led to an increase in the shrimp gut’s total mucosal surface [51]. Moreover, for *Sargassum*, the authors of [18] concluded that the diet supplemented with 0.5% *S. filipendula* (dry biomass) significantly enlarged the surface area of the gut absorption epithelium of *L. vannamei*. According to [52], the dietary addition of *Gracilaria lemaneiformis* at 2% dry biomass improved integrity of GI tract. It enlarged the length of microvilli cells in the gut absorption epithelium of *L. vannamei*. Likewise, the dietary addition of 2% *U. pinnatifida* (dry biomass) enlarged the length of intestinal villi in *P. monodon* juveniles [53].

Shrimp has a deficiency in the adaptive/specific immune system and depends on the innate/nonspecific immunity for protection against diseases and environmental changes [45]. Feed additives can stimulate both the humoral and cellular contents of the innate immune system [54]. Activities of lysozyme, total hemocytes, AP, SOD, PO, and TNO are vitally important nonspecific immune parameters reflecting the innate immune capacity [40]. The present study revealed that the dietary administration of both nucleotides and *Sargassum* significantly increased all the nonspecific immune responses with different levels of improvement. The superior results were in favor of T4. The results of this study are in agreement with many previous studies, both in terms of the positive effect of nucleotides [9,40,50,55] or *Sargassum* [25,31,56]. For nucleotides, shrimps fed dietary nucleotides-supplemented diets showed significantly higher lysozyme activities, T-SOD, and T-NOS when dietary nucleotides added at levels from 60 to 120 mg/kg, and activities decreased when the level of addition increased to 1200 mg/kg [50]. Similarly, the authors of [40] manifested that shrimp fed nucleotides-rich yeast diet at 50 g/kg indicated higher PO and lysozyme activities than the control group of *L. vannamei*. The administration of dietary nucleotides increased respiratory burst activity, PO activity, and THC [37].

For *Sargassum*, flavonoids are one of the compounds that play a significant role in enhancing the immune system. In this regard, in [25] the authors concluded that shrimp immersed in 150 ppm *Sargassum* extract showed proliferation of hemocyte and other hemolymph parameters. Schleder et al. [57] stated that the dietary addition of 4% of *U. pinnatifida* improved PO activity in *L. vannamei*. Ghaednia et al. [7] found that the content of total hematocytes count (THC), phagocytic activity (PA), differential hemocyte count (DHC), total plasma protein, bactericidal activity, and bacterial clearance efficiency increased significantly in *F. indicus* immersed in hot seawater containing *Sargassum glaucescens* extracts at 300 and 500 mg/L compared with the control. Phagocytic index, phagocytosis %, ABPC increased in shrimp fed with fucoidan and white spot syndrome virus (WSSV) infected 2.84-, 1.66-, and 1.55-fold compared with the control [8]. *S. polycystum* exhibited the best antimicrobial activity and the best natural immunostimulant compared to other *Sargassum species* (*S. oligocystum*, *S. crassifolium*, *S. cristaefoliumas*) against tested aquaculture microbial diseases [19].

Environmental stress negatively affects the shrimp immune system. Stressors increase shrimp vulnerability to diseases, probably due to long-lasting raised cortisol content, leading to immunosuppression [21]. Le Moullac and Haffner [58] observed that *P. stylirostris* showed decreased THC after exposure to ammonia content at 3 mg/L. The effects of low water temperature on the immune system of *L. vannamei* have not been studied yet. However, increasing water temperature from 27 to 33 °C significantly increased the total hemocyte count in *L. vannamei* hemolymph infected orally with WSSV, resulting in 100% survival [59]. However, the higher temperature might increase *Vibrio* bacteria populations in shrimp farms [60].

Shrimp diseases caused by viruses, particularly WSSV, and cold-water tolerance are the highest shrimp aquaculture challenges. A good understanding of shrimp immune response may help control these challenges [61]. The modulation of the immune response in shrimp farming through various marine seaweeds and/or prebiotics has become the main direction of scientific studies [40,62,63]. Studies on the relationship between nucleotides/seaweeds-enriched diets and gene expression in shrimps are minimal, whereas the effects of feed additives on gene expression under low water temperature have not been studied yet. Therefore, one of the main objectives of this study was to address this challenge. The present study findings showed that nucleotides/seaweeds-supplemented diets upregulated the relative expression of cMnSOD, Penaeidin4, and HSP70 genes, while TCTP was downregulated. The results agreed with the previous study for *Litopenaeus vannamei* fed *Aloe vera* [63], who reported similar findings in the immune-related genes (SOD, HSP70, penaeidin4, and TCTP). The addition of nucleotides in shrimp feeds displayed the maximum values of alkaline phosphatase and lysozyme relative genes expression when *L. vannamei* were fed 30 g/kg nucleotides-rich yeast supplementation compared with all treatments [40].

Heat shock protein (HSP70) is an accessory protein that has a vital role in the immune response as cheaper ones and inducers of proinflammatory cytokines secretion [64]. In *P. vannamei* shrimp, HSP70 has been documented [65]. It existed at low concentrations in many cells under stressful conditions, irrespective of their cell cycle stage [63]. The infection with bacteria or viruses caused an elevation in HSP70 level in *P. vannamei* [66].

Some plant compounds such as sodium alginate [67], *Panax ginseng* extract [68], *Rubus coreanus* extracts [69], *A. vera* [63] enhanced *SOD* gene expression in different species of shrimp. Additionally, environmental stressors such as abrupt changes in water temperature increased *MnSOD* gene expression. González-Ruiz et al. [70] concluded that high water temperature and hypoxia exhibited a synergistic effect in the upregulation on gene expression of *mMnSOD* in both gills and hepatopancreas. On the other side, the upregulated production of *MnSOD* gene expression in shrimp was related to pathogens infection and defense processes, as detected in *P. monodon* challenged with *V. harveyi* [71].

Microbial infections seem to be the main inducer of *Penaeidin4* gene expression [72], especially against Gram-positive, not Gram-negative bacteria [73]. Nevertheless, Wang et al. [74] discovered that *penaeidin4* was upregulated in *P. vannamei* challenged with Gram-negative bacteria like *V. harveyi*. The same trend was detected in *F. indicus* infected with *V. parahaemolyticus* [75]. On the other hand, some nutritional immunostimulants had an influential role in *Penaeidin4* gene expression. An increase in *Penaeidin4* gene expression due to the dietary addition of *A. vera* [63,76] was detected.

Downregulation of *TCTP* might be attributed to low water temperature. *TCTP* played essential multifunctional roles in cell growth [77], eukaryotes [71], and cell death pathway in shrimp hemocytes [78]. Bangrak et al. [78] stated that *TCTP* gene expression was decreased in *P. monodon* infected with a high WSSV burden. However, a study carried out by [61] concluded that *TCTP* was upregulated after WSSV challenge test.

## 4. Materials and Methods

### 4.1. Experimental Site

This experiment was carried out in El-Max Research Station, National Institute of Oceanography and Fisheries (NIOF), Alexandria, from 15 December 2019–15 February 2020. Juvenile whiteleg shrimp, *Litopenaeus vannamei* were purchased from a private shrimp farm and acclimatized to high saline water (salinity: 33 ± 0.5 ppt) that came from deep wells (100 m depth) for two months. Shrimp were fed the artificial formulated feeds purchased from ALLER Aquafeed Company (6 October City, Egypt) for two months before starting the current study. ALLER Aquafeed proximate composition was 38% crude protein, 7.5% crude lipids, 4.4% fiber, 4100 kcal/kg gross energy, and 9% moisture. This study was performed in 12 circular fiberglass tanks, each of 2 m^3^ water volume, representing the four experimental treatments in triplicate.

### 4.2. Experimental Shrimp and Culture Condition

Apparent healthy juvenile whiteleg shrimp, *Litopenaeus vannamei* with an average initial body weight of 12.05 ± 0.03 g and an average initial total length of 11.83 ± 0.25 cm were used to perform this experiment. A total of 480 shrimp were stocked in the 12 experimental tanks located outdoor at a density of 40 shrimps per tank (20 pcs/m^3^) representing the four experimental treatments in triplicate. Each treatment (120 shrimp) was performed in three separate tanks and each tank contained 40 shrimp representing one replicate. Tanks were supported with continuous aeration coming from the air blower. To avoid an increase in the tank water temperature due to using of somewhat warmer water (around 20–21 °C) coming from deep wells (100 m depth), 10% daily water exchange was operated to keep the water temperature below 16 °C. Thus, to simulate shrimp farms’ natural situation during the winter season and water temperature in the experiment shrimp tanks were left without human control.

### 4.3. Tested Feed Additives (NucleoforceFish™ and Sargassum polycystum)

NucleoforceFish™ was obtained from Bioiberica^®^ Spain (Barcelona, Spain). The proximate composition of NucleoforceFish™ was composed of crude protein 35.9%, crude ash 8.5%, crude fiber 0.1%, non-protein nitrogen (NNP) 4.7%, free nucleotides 34% from inactivated yeast 92 extract, 80% pyrimidine, and 20% purine. The brown seaweeds, *S. polycystum* were collected from Hurghada, Red Sea, Egypt. The *S. polycystum* were washed with fresh water and dried at 27 ± 3 °C for two days, before drying in an oven at 45 °C for 24 h, and then ground into powdered seaweed flour. Proximate analysis of *S. polycystum* is illustrated in Table 4.

### 4.4. Experimental Treatments

Shrimp were fed four experimental diets by hand, as illustrated in Table 5. Treatments were tested as follows:

T1: Basal diet; free from tested additives (control).
T2: Basal diet supplemented with 500 mg/kg nucleotides (as recommended by [79])
T3: Basal diet supplemented with 500 mg/kg *S. polycystum* powdered.
T4: Basal diet supplemented with 500 mg/kg nucleotides and 500 mg/kg *S. polycystum* powdered.

Feed ingredients were finely grounded, sieved, homogenized, and mixed using electric kitchen mixers. Then, the experimental diets were manufactured using an electric kitchen meat grinder (Moulinex ME605131—HV8—1600 W, Paris, France) after the addition of hot water. Feed pellets were first dried at room temperature in a well-ventilated laboratory for 3 h. The feed pellets (2 mm diameter * around 5 mm length) dried at 45 °C for 12 h, and then stored at −20 °C until use. The tested diets were not less than 36.1% crude protein and 4.76% crude lipid. Feed ingredients, formulation and proximate composition (%, on fresh/dry matter (FM/DM) bases) of the tested diets is illustrated in Table 5. Shrimp were fed the experimental diets ad libitum, four times daily (during daylight hours), for 56 days.

### 4.5. Measured Traits

#### 4.5.1. Water Analysis

Water temperature, salinity, pH, and dissolved oxygen were monitored by SensoDirect 150 MultiMeter (Lovibond, Tintometer Limited, Amesbury, UK) once every three days with a total of 60 samples per group. Water temperature was measured in the early hours of the morning. Total ammonia nitrogen (TAN) was monitored by HANNA HI96715-11 Ammonia Medium Range photometer (HANNA, Nusfalau, Romania) once every week with a total of 24 samples per group. Unionized ammonia (NH3) was determined from the pre-estimated TAN, temperature, and pH values of the same tested group [80]

#### 4.5.2. Growth Performance Parameters

Weight gain (WG), average daily gain (ADG), specific growth rate (SGR), condition factor, and survival rate were measured as follows:

Weight gain (g/fish): WG = *W_t_* − *W*_0_

where *W*_0_: the initial mean weight of shrimp in grams. *W_t_*: the final mean weight of shrimp in grams.

Average daily gain (g/shrimp/day): ADG = *W_t_* − *W*_0_*/n*

where, *n*: experimental days (56 day).

Specific growth rate (%/day): SGR = 100 × (ln *W_t_* − ln *W*_0_)/*n*

where, ln: natural logarithm.

Survival rate (%) = 100 × (initial number of shrimp/final number of shrimp)

Condition factor = 100 × [*W_t_* (g)/*L_t_*^3^ (cm)]

where, *L_t_*: Final length of shrimp (cm).

#### 4.5.3. Feed and Nutrients Utilization Parameters

Feed intake (g/shrimp): this is the total quantity of feed given each shrimp during the experimental days per gram.

Feed conversion ratio (FCR) = dry matter feed intake (g)/shrimp weight gain (g).

Protein efficiency ratio (PER) = shrimp gain/protein intake.

Protein productive value (PPV %) =100 × (*P_t_* − *P*_0_/protein intake (gm).

where, *P*_0_: protein content in shrimp whole-body at the start of the experiment. *P_t_*: protein content in shrimp whole-body at the end of the experiment.

Energy utilization (EU, %) = 100 × (energy gain (*K_cal_*/100 g)/energy intake (*K_cal_*/100 g)).

#### 4.5.4. Shrimp and Feed Analytical Methods

At the beginning and end of the experiment, shrimp and feed samples were analyzed to determine the proximate composition of moisture, crude protein, ether extract, fiber, and ash contents [81]. A representative sample of shrimp on stocking date was subjected to body chemical analysis. Whole shrimp content of moisture, crude protein, and ether extract contents on a fresh/dry matter basis were determined according to [81] methodology.

#### 4.5.5. Nonspecific Immune Responses

I. Total hemocyte count (THC): the THC was estimated based on the technique of [82]. Before hemolymph collection, shrimp were anesthetized using clove oil at 0.2 mL/L. A hemocytometer (Hausser Scientific, Horsham, UK) used to count hemocytes and the result was calculated based on the equation: Total hemocytes (cells/mL) = average cells number × 10^4^ × dilution factor

II. Phagocytosis assay: phagocytotic assay was determined based on the slightly modified technique of [83]. The phagocytic percentage (PP) and phagocytic index (PI) were calculated as follows:

The phagocytic percentage (PP) = number of phagocytic hemocytes/number of hemocytes observed × 100

Phagocytic index (PI) = number of bacteria *C. albicans* ingested by hemocytes/number of phagocytic hemocytes

III. Determination of lysozyme activity: lysozyme activity was determined following the turbidimetric method [84]. Briefly, 0.2 mg/mL lyophilized *Micrococcus lysodekticus* (Sigma, St Louis, MO, USA) was used as the substrate in pH 5.75 phosphate buffer. In total, 50 µL of hemolymph were added to 3 mL of the bacterial suspension. Using spectrophotometer at 540 nm (nanometer), the absorbance value was measured after mixing (*A*_0_) and after 30 min incubation at 37 °C (*A*). Each reduction in the absorbency with 0.001/min was expressed as one unit of lysozyme activity. The following equation was used to determine the value of lysozyme activity.

Lysozyme activity = (*A*_0_ − *A*)/*A*.

IV. Phenoloxidase (PO) activity: the method used for measuring the phenoloxidase activity was based on the spectrophotometrically published modified protocol [85]. This method depends on recording the formation of dopachrome produced using l-DOPA (l-dihydroxyphenylalanine). The mixture of hemolymph (0.1 mL) and anticoagulant buffer composed of 450 mM NaCl, 100 mM glucose, 26 mM citric acid, 30 mM sodium citrate, pH 4.6 (0.9 mL) was washed three times with shrimp saline (water salinity = 33 ± 0.5 ppt), homogenized and centrifuged at 1500 rpm at 4 °C for 10 min. The supernatant was wasted and to the pellet a cacodylate-citrate buffer solution (0.01 M sodium cacodylate, 0.45 M sodium chloride, 0.01 M calcium chloride, and 0.26 M magnesium chloride; pH 7.0) was added, then homogenized, and the suspension was centrifuged at 10,000 rpm at 4 °C for 20 min. Then, 200 μL of hemocyte lysate was mixed with the 200 μL of 0.25 % trypsin in cacodylate buffer, followed by 200 μL l-dihydroxyphenylalanine at 4 mg/mL as substrate. The enzyme activity (absorbance of dopachrome) was measured spectrophotometrically at a wavelength of 492 nm. The phenoloxidase activity measurement was estimated as the increase in the optical density (OD) per minute per milligram of protein.

V. Superoxide dismutase activity (SOD): the SOD activity was measured according to the method of Nishikimi et al. [86] by its ability to inhibit superoxide radical-dependent reactions using a Ransod kit (Randox, Crumlin, UK). The methodology depends on the formation of red formazan dye as the reaction of superoxide radicals with 0.025 mM INT (dissolved in 50 mM CAPS and 0.94 mM EDTA). Superoxide, xanthine oxidase, and uric acid are produced from the xanthine. The plasma was produced by the centrifuging process of the hemolymph–anticoagulant mixture at 3000 rpm for 10 min. Plasma was removed, and the pellet was resuspended and centrifuged again with 3 mL of 0.9 % NaCl. The supernatant was wasted, and the pellet was resuspended with 2 mL of triple distilled water. Hemocytes volume of 50 μL was placed in each well, with 96-well plate containing 200 μL of the reaction mixture. A total of 50 μL of xanthine oxidase was added to each well. The absorbance was measured at 505 nm and 37 °C. After adding xanthine oxidase, the absorbance reading was measured at 0.5 and 3 min. The amount required to inhibit 50% of xanthine reduction specific activity was expressed as one unit of SOD (U/mL).

VI. Bactericidal activity: the bactericidal activity was determined as reported earlier [85]. First, shrimp serum was separated from the blood sample of each treatment. Then serum was diluted in 2.6% NaCl solution at the subsequent ratios, 1:2, 1:4, 1:8, 1:16, and 1:32, then 0.5 mL of each diluted serum sample was used for the bactericidal activity assay. The control sample with 0.1 mL of NaCl was used in the assay. A total of 0.1 mL of *Vibrio harveyi* suspension (8.2 × 10^6^ CFU/mL) was added to each serum dilution and control sample, incubated at room temperature for 3 h before the bacterial counting. The bactericidal activity value was recorded from a serum dilution that could decrease the number of *V. harveyi* by 50% compared with the CG group.

VII. The shrimp serum’s protein concentration: it was determined using bovine serum albumin as the standard based on the Bradford Method [87].

VIII. Acid and alkaline phosphatase activities: this parameter was determined based on the method described by [88], with minor alterations. To examine the effect of pH, 80 mM citric-sodium citrate buffer in the range of 3.0–6.0 pH, 80 mM Tris-HC1 buffer in the range of 7.0–8.0 pH, and 80 mM glycine-NaOH buffer in the range of 8.5–12.0 pH were used. Standard assay conditions were 80 mM glycine-NaOH buffer at pH 10.5 (ALP), 80 mM citric-sodium citrate buffer at pH 4.5 (AcP) and 8 mM p-nitrophenyl phosphate disodium salt, and 100 µL of the volume of the extract. The final volume was 1.2 mL, and the incubation temperature 30 °C. To determine the effects of metals, we used 100 p.1 of a metal solution at a final level of 1 mM in the medium. The reaction was ended by adding 5 mL of 0.1 N NaOH. The absorbance of the released p-nitrophenol was measured at a wavelength of 405 nm. Results are expressed as U/g (1 U = 1 µmol p-nitrophenol/min) [88].

#### 4.5.6. Histological Examination

The hepatopancreas and intestinal tract of *L. vannamei* were taken from the different groups. The samples were fixed in 4% buffered formalin for 24 h. Then, tissues were dehydrated by passing a series of alcohol solutions (70%, 85%, and 98%), and finally the tissues were embedded in paraffin. The histological sections of 4–5 µm (Leica, Germany) were stained with hematoxylin and eosin (H&E) for general morphological purposes and photographically documented using an Olympus BX50 microscope (Japan) [89].

#### 4.5.7. Hemolymph and Gene Expression Analysis

I. Hemolymph: hemolymph sample was drawn from the ventral sinus of shrimp from each treatment, using a 1 mL sterile syringe containing a 1 mL of ice-cold anticoagulant solution (450 mM NaCl, 100 mM glucose, 26 mM citric acid, 30 mM sodium citrate, pH 4.6). The hemolymph samples collected from four shrimps from each tank were mixed as one representative sample and three replicates. Samples were centrifuged at 3000 rpm for 10 min at 4 °C and the supernatant fluid was directly stored at −80 °C. The collected hemolymph samples were immediately frozen in liquid nitrogen, and stored at −80 °C for assay of gene expression [89,90].

II. RNA Extraction and cDNA Synthesis: according to the manufacturer’s manual, total RNA was extracted from hemolymph samples using Trizol reagents (Invitrogen, UK). The reverse transcription (RT-PCR) was performed as described by [91] using SYBR green method in an iQ5 iCycler thermal cycler (Bio-Rad, Hercules, CA, USA). The reactions were set on a 96-well plate by mixing 1 μL of diluted (1/20) cDNA, 5 μL of 2× concentrated iQ TM SYBR Green Supermix (Bio-Rad) as a fluorescent intercalating agent, 0.3 μM forward primer and 0.3 μM of the reverse primer. The sequences of specific primers used for *cMnSOD, TCTP, Penaeidin4*, and heat shock protein 70 (*HSP70*), respectively, are presented in Table 6. The β-actin was used as the housekeeping gene. The real-time PCR program was adjusted at 95 °C for 1 min, followed by 40 cycles at 95 °C for 15 s, 60 °C for 15 s, 72 °C for 45 s, and one step of 95 °C for 10 s. To obtain the melting curves, the temperature increased from 65 to 95 °C (0.5 °C/s) to denature the double-stranded DNA. The relative mRNA expressions were calculated by the comparative Ct method (2^−ΔΔCt^) [92].

### 4.6. Statistical Analysis

The obtained data were checked for normality by analyzing distribution frequency within the histogram. An arcsine transformation was used before processing percentage data. Means and standard error of mean (mean ± SEM) for each tested parameter were calculated. The data were subjected to statistical analysis, one-way analysis of variance (ANOVA) using SPSS Version 22 to assess the effect of dietary addition of *S. polycystum* and/or nucleotides on survival, growth performance, whole-body chemical analyses, feed utilization, oxidative stress, immune responses, and immune-related genes. Differences between means were compared using [95] multiple range tests using SPSS 22 for Windows (Standard Version 22 SPSS Inc. Chicago, IL, USA). Impacts with a probability of *p* ≤ 0.05 were considered statistically significant. The figures were created using Graph Pad Prism 6 (Graph Pad Prism v6.0, San Diego, CA, USA).

## 5. Conclusions

It was suggested that the synergistic effects of *S. polycystum* and nucleotides improved growth performance, feed utilization efficiency, immune response, histomorphology of hepatopancreas and intestinal tract, and cold-tolerant gene expression (upregulated the relative expression of *cMnSOD*, *Penaeidin4*, and *HSP70* genes, while *TCTP* gene was downregulated) in *L. vannamei.* Hence, *S. polycystum* and nucleotides could be encompassed during the formulation of aquafeeds to manipulate disease/stress via dietary management.

## Figures and Tables

**Table 1:** Water quality parameters of the experimental tanks stocked with whiteleg shrimp (*Litopenaeus vannamei*) fed powdered *Sargassum polycystum*- and nucleotides-supplemented diets during the winter season.

Parameters	Treatments *			
	T1	T2	T3	T4
Water salinity, ppt	32 ± 0.05	32 ± 0.04	32 ± 0.08	32 ± 0.06
Temperature, °C	13.33 ± 0.07	13.27 ± 0.07	13.33 ± 0.03	13.43 ± 0.03
pH	8.20 ± 0.01 ^a^	8.14 ± 0.05 ^ab^	8.06 ± 0.04 ^b^	8.12 ± 0.01 ^ab^
DO, ppm	5.97 ± 0.09 ^c^	6.37 ± 0.03 ^b^	6.40 ± 0.06 ^b^	6.67 ± 0.09 ^a^
TAN, ppm	0.48 ± 0.02 ^a^	0.39 ± 0.01 ^b^	0.38 ± 0.01 ^b^	0.24 ± 0.02 ^c^
NH3, ppm	0.0176 ^a^	0.0125 ^b^	0.0102 ^bc^	0.0075 ^c^

* Treatments; T1 = Control; T2 = 500 mg/kg Nucleotides; T3 = 500 mg/kg *Sargassum polycystum* powdered; T4 = 500 mg/kg Nucleotides + 500 mg/kg *Sargassum polycystum* powdered. Means within the same row with different superscript letters (a–c) are significantly different (*p* < 0.05).

**Table 2:** Growth performance indices, survival, feed utilization indices, and whole-body proximate composition of whiteleg shrimp (*Litopenaeus vannamei*) fed powdered *Sargassum polycystum*- and nucleotides-supplemented diets during the winter season.

Parameters	Treatments *			
	T1	T2	T3	T4
**Growth performance indices and survival**				
Initial weight (g)	12.04 ± 0.02	12.05 ± 0.01	12.06 ± 0.02	12.02 ± 0.02
Final weight (g)	15.28 ± 0.01 ^d^	15.62 ± 0.04 ^b^	15.46 ± 0.02 ^c^	15.82 ± 0.02 ^a^
Weight gain (g)	3.24 ± 0.02 ^d^	3.57 ± 0.02 ^b^	3.40 ± 0.04 ^c^	3.81 ± 0.02 ^a^
ADG (g/shrimp/day)	0.058 ± 0.0 ^d^	0.064 ± 0.00 ^b^	0.061 ± 0.00 ^c^	0.068 ± 0.00 ^a^
SGR (%/shrimp/day)	0.425 ± 0.001 ^d^	0.463 ± 0.002 ^b^	0.444 ± 0.005 ^c^	0.491 ± 0.003 ^a^
Condition factor	0.583 ± 0.003 ^c^	0.700 ± 0.006 ^b^	0.690 ± 0.006 ^b^	0.753 ± 0.003 ^a^
Survival rate (%)	75.00 ± 1.44 ^b^	82.50 ± 1.44 ^a^	80.00 ± 1.44 ^a^	84.17 ± 0.83 ^a^
**Feed utilization indices**				
FCR	3.91 ± 0.043 ^a^	3.14 ± 0.051 ^c^	3.42 ± 0.035 ^b^	2.78 ± 0.021^d^
PER	0.637 ± 0.009 ^d^	0.793 ± 0.015 ^b^	0.727 ± 0.009 ^c^	0.897 ± 0.009 ^a^
PPV, %	46.25 ± 1.37 ^b^	46.27 ± 2.46 ^b^	37.80 ± 1.22 ^c^	64.74 ± 1.07 ^a^
Energy utilization (EU %)	26.77 ± 0.58 ^b^	25.52 ± 1.48 ^b^	19.54 ± 0.88 ^c^	35.14 ± 0.82 ^a^
**Whole-body proximate composition**				
Dry matter (%)	20.43 ± 0.02 ^c^	21.16 ± 0.01 ^b^	21.79 ± 0.02 ^a^	21.73 ± 0.08 ^a^
Crude protein (%)	73.45 ± 0.32 ^a^	70.20 ± 0.59 ^b^	68.92 ± 0.34 ^c^	73.35 ± 0.20 ^a^
Ether extract (%)	3.19 ± 0.12 ^a^	2.58 ± 0.14 ^b^	2.07 ± 0.11 ^b^	2.54 ± 0.26 ^b^
Ash (%)	16.05 ± 0.31 ^a^	14.55 ± 0.37 ^bc^	13.88 ± 0.35 ^c^	15.04 ± 0.18 ^ab^
Fibre (%)	4.86 ± 0.03 ^d^	5.24 ± 0.05 ^c^	5.46 ± 0.05 ^b^	5.63 ± 0.04 ^a^
Carcass energy (Kcal/100g)	444.42 ± 0.97 ^a^	420.26 ± 3.55 ^b^	408.27 ± 2.83 ^c^	437.68 ± 2.84 ^a^

* Treatments; T1 = Control; T2 = 500 mg/kg Nucleotides; T3 = 500 mg/kg *Sargassum polycystum* powdered; T4 = 500 mg/kg Nucleotides + 500 mg/kg *Sargassum polycystum* powdered. Means within the same row with different superscript letters (a–d) are significantly different (*p* < 0.05).

**Table 3:** Nonspecific immune responses of whiteleg shrimp (*Litopenaeus vannamei*) fed powdered *Sargassum* *polycystum*- and nucleotides-supplemented diets during the winter season.

Parameters	Treatments *			
	T1	T2	T3	T4
Total hematocytes count (cells/mm^3^)	19.00 ± 0.58 ^d^	27.00 ± 0.58 ^b^	22.33 ± 0.88 ^c^	32.33 ± 1.45^a^
Phagocytosis (%)	22.20 ± 0.55 ^d^	29.30 ± 0.61 ^b^	26.63 ± 0.44 ^c^	33.37 ± 0.64 ^a^
Phagocytic index	2.57 ± 0.20 ^d^	4.13 ± 0.27 ^b^	3.50 ± 0.12 ^c^	5.13 ± 0.15 ^a^
Total protein (mg m L^−1^)	5.77 ± 0.43 ^c^	11.63 ± 0.55 ^a^	7.97 ± 0.42 ^b^	12.93 ± 0.43 ^a^
Acid phosphatase activity (U L^−1^)	15.97 ± 0.72 ^c^	23.17 ± 0.75 ^b^	21.40 ± 0.72 ^b^	25.77 ± 0.47 ^a^
Alkaline phosphatase (U L^−1^)	6.33 ± 0.72 ^c^	14.70 ± 1.47 ^b^	8.80 ± 0.15 ^c^	20.37 ± 0.50 ^a^
Lysozyme activity (U L^−1^)	59.57 ± 1.07 ^d^	78.70 ± 2.57 ^b^	68.10 ± 1.63 ^c^	97.17 ± 2.30 ^a^
Phenoloxidase activity (U/min/mg)	15.83 ± 1.28 ^d^	25.77 ± 0.76 ^b^	20.30 ± 0.52 ^c^	29.73 ± 0.50 ^a^
Superoxide dismutase activity (U/min/mg)	0.38 ± 0.02 ^d^	0.58 ± 0.02 ^b^	0.48 ± 0.02 ^c^	0.74 ± 0.04 ^a^
Total nitric oxide (μg/mL)	11.73 ± 0.75 ^d^	23.13 ± 0.74 ^b^	19.67 ± 0.88 ^c^	27.20 ± 0.93 ^a^

* Treatments; T1 = Control; T2 = 500 mg/kg nucleotides; T3 = 500 mg/kg *S. polycystum* powdered; T4 = 500 mg/kg nucleotides + 500 mg/kg *S. polycystum* powdered. Means within the same row with different superscript letters (a–d) are significantly different (*p* < 0.05).

**Table 4:** Proximate nutritional analysis *of Sargassum polycystum* C. Agardh (g.100 g^−1^ DW of seaweed).

Parameter	%, DM
Protein (%)	14.99
Ether extract (%)	7.74
Fiber (%)	16.98
Carbohydrate (%)	24.93
Ash content (%)	28.57

**Table 5:** Feed formulation (g/kg) and proximate composition (%, fresh matter, FM and dry matter, DM) of the experimental shrimp diets supplemented with nucleotides and brown powdered seaweed, *Sargassum polycystum*.

Ingredients (g/kg)	Experimental Diets			
	T1	T2	T3	T4
Soybean meal	340	340	340	340
Wheat meal	150	150	150	150
Fish meal	112	112	112	112
Corn gluten, 60% CP	81	80.5	80.5	80
Rice bran	80	80	80	80
Corn grain	60	60	60	60
Shrimp meal	50	50	50	50
Meat meal	50	50	50	50
Vitamin and mineral premix	30	30	30	30
Dicalcium phosphate	20	20	20	20
Soybean oil	10	10	10	10
Fish oil	8	8	8	8
Lecithin	5	5	5	5
Choline chloride	2	2	2	2
Cholesterol	1	1	1	1
Vit. C	1	1	1	1
Nucleotides	0	0.5	0	0.5
*Sargassum polycystum*	0	0	0.5	0.5
Total	1000	1000	1000	1000
**Proximate composition, %/FM/DM ^1^**				
DM, (%)	90.55/100	90.42/100	90.45/100	90.58/100
CP, (%)	36.34/40.13	36.41/40.27	36.17/39.99	36.28/40.05
EE, (%)	4.56/5.04	4.51/4.99	4.54/5.02	4.55/5.03
CF, (%)	2.36/2.61	2.32/2.57	2.37/2.62	2.34/2.58
Ash, (%)	11.22/12.39	11.18/12.36	11.30/12.49	11.26/12.43
NFE, (%) ^1^	45.52	45.58	45.62	45.57
Gross energy (MJ/kg diet) ^2^	18.25	18.26	18.22	18.24
Gross energy (kcal/kg) ^3^	4350.84	4352.77	4343.66	4348.68
Energy/protein (kcal/g protein)	119.73	119.55	120.09	119.86
Ca, (%)	2.15	2.11	2.19	2.17
Total P, (%)	1.53	1.52	1.56	1.54
Available P, (%)	1.25	1.23	1.26	1.25

^1^ NFE (nitrogen-free extract) = 100 − (crude protein + ether extract + crude fiber + ash). ^2^ Gross energy (MJ/kg diet) = (% crude protein × 23.6) + (% crude lipids × 39.5) + (% NFE × 17.3). ^3^ Gross energy (Kcal/kg diet) = (% crude protein × 5.64) + (% crude lipids × 9.44) + (% NFE × 4.11).

**Table 6:** Specific primers used for qPCR amplification of housekeeping and immune-related genes of *P. vannamei.*

Genes	Primers	Sequence (5’–3’)	References
*SOD*	SOD-F	ATCCACCACACAAAGCATCA	[74]
	SOD-R	AGCTCTCGTCAATGGCTTGT	
*TCTP*	TCTP-F	CAATGGACCCTGATGGC	[61]
	TCTP-R	GCTTCTCCTCTGTTAGACCGTAT	
*Penaeidin4*	Pen4-F	GCCCGTTACCCAAACCATC	[74]
	Pen4-R	CCGTATCTGAAGCAGCAAAGTC	
*HSP70*	HSP70 F	GGCAAGGAGCTGAACAAGTC	[93]
	HSP70 R	TCTCGATACCCAGGGACAAG	
*β-actin*	β-actin-F	CCACGAGACCACCTACAAC	[94]
	β-actin-R	AGCGAGGGCAGTGATTTC	

## Data Availability

Data is contained within the article.
